# Micro-Dosing of Fine Cohesive Powders Actuated by Pulse Inertia Force

**DOI:** 10.3390/mi9020073

**Published:** 2018-02-07

**Authors:** Hongcheng Wang, Ting Zhang, Miaomiao Zhao, Rangrang Chen, Liqun Wu

**Affiliations:** School of Mechanical Engineering, Hangzhou Dianzi University, Hangzhou 310018, China; 172010042@hdu.edu.cn (T.Z.); 152010075@hdu.edu.cn (M.Z.); 162010046@hdu.edu.cn (R.C.); wuliqun@hdu.edu.cn (L.W.)

**Keywords:** fine powder, micro-dosing, piezoelectric actuator, nozzle, pulse inertia force

## Abstract

Micro-dosing of fine cohesive powders is the key technology in additive manufacturing and especially in high-potency active pharmaceutical ingredients (HPAPI). However, high accuracy micro-dosing (<5 mg) of fine cohesive powder is less trivial and still remains a challenge because it is difficult to eliminate the aggregation phenomena caused by the strong interparticle cohesive forces (in small capillaries). This paper presents a novel micro-dose method of fine cohesive powders via a pulse inertia force system. A piezoelectric actuator is used to provide a high enough pulse inertia force for a tapered glass nozzle and drive powder particles in the nozzle to be discharged from the nozzle orifice with the help of particle self-gravity. The nozzles with outlet diameters in the range of 100–2000 µm were fabricated via a glass heating process. The α-lactose monohydrate powder is used as the micro-dosing powder. The influences of the tapered nozzle outlet diameter, amplitude of the applied pulse voltage, and angle of the nozzle axis on micro-dosing mass are researched. The minimum mean dose mass is 0.6 mg for a single pulse inertia force. The coefficient of variation of dose mass, which represents the micro-dosing stability, can be controlled below 5% when the dose mass is relatively small.

## 1. Introduction

Micro-dosing of fine powders can be applied in additive manufacturing [1] and in the pharmaceutical industries, typically, in selective laser sintering (SLS) [2], three-dimensional printing (3DP) [3], and high-potency active pharmaceutical ingredients (HPAPI). For example, in HPAPI [4], the dosing of only a few mg (or less) of active pharmaceutical ingredient (API) powders in a drug product is needed. However, these API powders (e.g., a-lactose monohydrate powder) are made of very fine and sticky particles [5]. The size of individual particles is reduced below several microns. The interparticle cohesive forces begin to play a major role in the bulk powder behavior. Van der Waals forces are the main cohesive forces between fine particles. The strong interparticle adhesion forces cause the formation of aggregates [6,7], which may cause micro-dosing process unstability and inaccuracy. So, accurate micro-dosing of these fine cohesive powders appears to be an extremely critical step in practice. 

In recent years, many powder dosing techniques, such as pneumatic methods [8,9] and vibratory methods, have been emerging in the above research fields. In a pneumatic powder dosing system, the compressed gas is used to form gas-powder streams and the powder will be discharged out of a multi-channel nozzle. However, the powder is easy to form dense-phase [10] and aggregates in the nozzle. Another drawback of the pneumatic method is that the powder convergence is low when the powder is ejecting out of the nozzle, and as a result, it is difficult to control the powder dosing location. The vibration methods mainly include an acoustic vibration feeder [11] and ultrasonic vibration feeders, depending on the vibration frequency. In an ultrasonic vibration feeder, small capillaries [12,13,14] with orifices having a diameter in the same order of magnitude as the particles are often used. The feed rate can be controlled, e.g., via the frequency or the orifice diameter. However, powders that are cohesive and of a low density tend to block the capillary tube owing to particle aggregates, which are not able to break under mechanical vibration [15,16]. Since gravitational forces are comparatively small, inter-particle forces become important [6]. Blocked or unstable flow may occur due to an improper capillary structure in the capillaries with different structures. 

Besides that, Besenhard et al. recently demonstrated that micro-feeding with a vibratory sieve system mounted on a chute [17,18] or a cylinder piston system [19] is possible. Feed rates of a few mg/s for very fine powders could be obtained. However, powder properties required special attention. Feed rates were shown to increase monotonically with the vibratory frequency and different powder properties can lead to significant variations in the feed rate. 

In sum, high accuracy micro-dosing (below 5 mg for a single pulse) of fine cohesive powder is less trivial and still remains a challenge due to the aggregation phenomena in small capillaries. To solve this problem, we developed a simple fine cohesive powder micro-dosing approach and the powder is discharged from a tapered glass capillary actuated by a hollow piezoelectric transducer (PZT) stack without any complex structure.

## 2. Materials and Methods 

### 2.1. Materials

**Figure 1:**
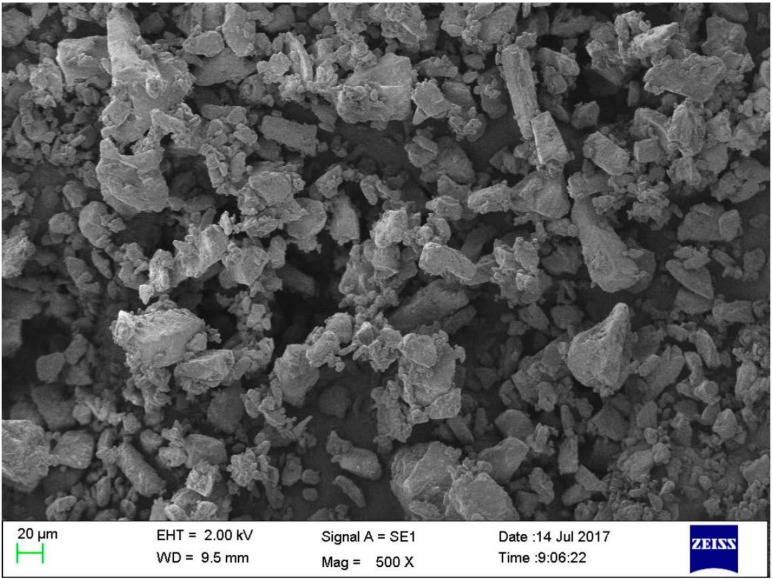
SEM images of RespitoseSV003 monohydrate powder.

In the experiment, the α-lactose monohydrate powder (RespitoseSV003) supplied by DFE Pharma (Goch, Germany) is used as the dosing fine cohesive powder. The micro morphology and characteristics were studied with SEM (EVO18, ZEISS, Germany), which shows that there are many small particles adhering to larger ones and most of the larger particles have prism shapes, as is shown in Figure 1. This micro-pattern indicates that the α-lactose monohydrate powder has strong cohesive forces between the particles. 

Properties of the powder are described in Table 1. The Angle of Repose (AoR) was determined using a glass funnel by an AoR determinator (FT-104B2, Beijing Zhongyiwancheng Technology Co., Ltd., Beijing, China). The aspect ratio (AR) is the ratio between *F_min_* and *F_max_* (Feret diameters), which describes the shape of particles. Its value can be between 0 and 1. The higher the value is, the more spherical the shape is. So, the low aspect ratio value of 0.69 shows that the particles of the powder are of an irregular shape, which may cause the aggregation phenomenon more seriously. The powder was stored at a relative humidity (RH) < 55% and a room temperature <25 °C.

### 2.2. Experiment Apparatus

**Figure 2:**
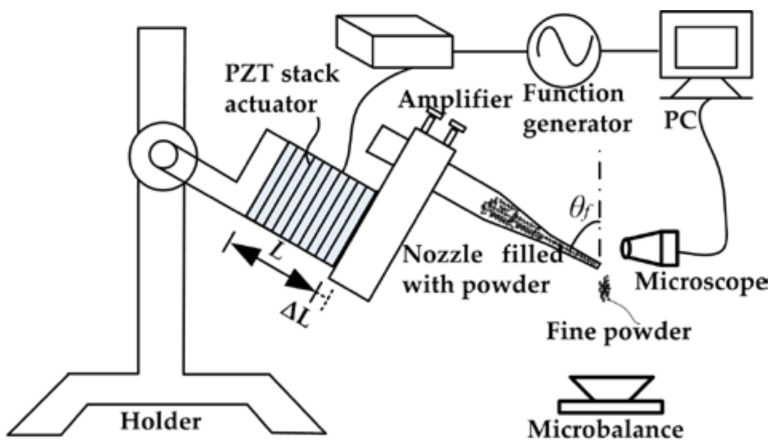
Apparatus for micro-dosing of fine cohesive powder actuated by pulse inertia force.

The pulse inertia force providing system for the fine cohesive powders micro-dosing system was designed, as is shown in Figure 2. The structure of the micro-dosing system mainly consists of five parts: a signal generating device, a PZT stack actuator, a tapered glass capillary nozzle, a microscopic system, and a powder dosing mass weighing device. The detailed instructions for the above five parts are described in the following.

**Figure 3:**
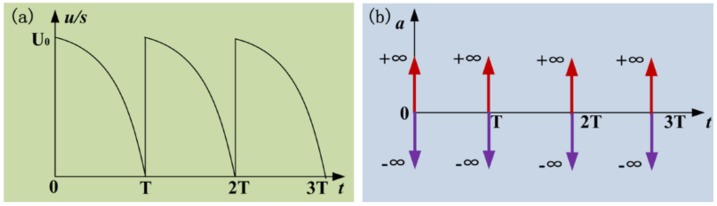
Driving signal: (**a**) voltage-time curve and (**b**) acceleration-time theoretical curve.

The driving voltage signal generating device consists of a function generator (DG1032Z, Rigol, Beijing, China) and an amplifier (PDS21, NanoMotions, Ronkonkoma, NY, USA). The function generator is used to produce an original driving signal, which is one quarter of a sine wave, as is shown in Figure 3a. For a single driving period, when the time of *t* is 0, the voltage sharply increases from zero to amplitude. When the time of *t* exceeds zero, the voltage slowly decreases from amplitude *U*_0_ to zero. The frequency *f_0_* of the driving signal can be controlled from 1 to 100 Hz. The amplifier is to used amplify the amplitude and current to a value large enough to drive the PZT stack actuator (PAL200VS25, NanoMotions, Shanghai, China). The amplitude *U_0_* can be controlled in the range of 0–110 V. The PZT stack actuator is constructed of several disc-shaped piezoelectric ceramic pieces, and the thickness is in the range of 0.02–1 mm. The upper surface is fixed with connector A, which is also fixed with a holder without any movement, while the lower surface is fixed with connector B. There is an approximate linearity between the applied voltage amplitude and the lower surface displacement of the actuator. So, the actuator will cause a larger displacement instantaneously and consequently provide a greater pulse inertia force for the micro-nozzle and particles inside when a higher driving voltage pulse is applied. 

**Figure 4:**
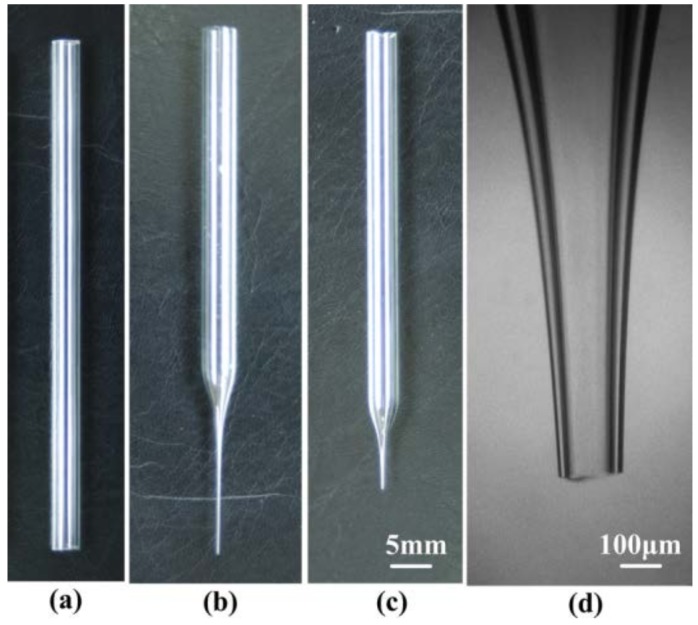
Fabrication of the borosilicate glass micro-nozzle: (**a**) borosilicate glass pipe; (**b**) micro-nozzle after being pulled; (**c**) micro-nozzle after being cut; (**d**) micrograph of micro-nozzle after being cut.

Glass material was chosen to produce the tapered glass capillary because of several advantages, such as good chemical resistance, smooth surface, ease of manufacture and observation, and low cost. The raw material is a borosilicate glass capillary (Beijing Zhengtianyi Scientific and Trading Co., Ltd., Beijing, China). The dimensions of the glass capillary are 7.8 mm, 5.4 mm, and 100 mm in external diameter, internal diameter, and length, respectively, as is shown in Figure 4a. A glass heating process was adopted to fabricate the micro-nozzle without complicated micro-fabrication technology and can be divided into two steps: (1) pulling a capillary to form a micro-nozzle with a straight outlet, as is shown in Figure 4b; and (2) forging the straight outlet to form a shrinkage one, as is shown in Figure 4c. The detailed fabrication process for the micro-nozzle has been presented in the literature [20]. The micro-nozzles with different outlet diameters (*d*) can be obtained by varying the control parameters of the voltage amplitude and the balance weights (the outlet diameter in this article means the inner diameter of the nozzle tip). The fabricated micro-nozzle with an outlet diameter of 100 μm is shown in Figure 4d.

The microscope system is used to observe the micro-dosing process. The powder dosing mass weighing device is used to record the dosing mass for each pulse inertial force.

### 2.3. Powder Dosing Principle

**Figure 5:**
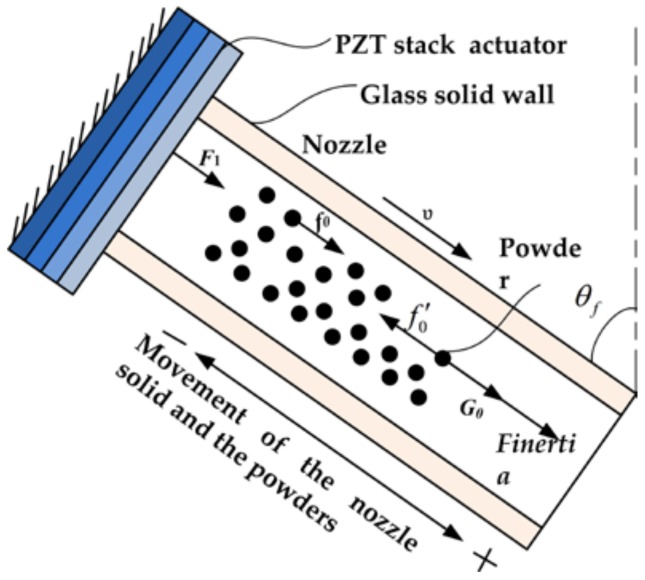
Driving principle for powder actuated by pulse inertia force.

The micro-dosing principle of fine cohesive powders actuated by pulse inertia force is shown in Figure 5. Because there is an approximate linearity between the applied voltage amplitude and the upper surface displacement of the actuator, the waveform of the driving voltage applied on the PZT stack actuator is the same as the displacement of the lower surface of the actuator. When being applied with a driving signal, the PZT actuator expends and exerts a driving force *F*_1_ on the solid wall of the glass nozzle.

When the time of *t* is 0, the voltage sharply increases from zero to amplitude. The acceleration of the lower surface of the actuator is +∞ firstly and −∞ secondly, as is shown in Figure 3b. During the process of the acceleration increasing to +∞, the glass solid wall and the powder exhibit a movement, along with the nozzle axis, to the outlet of nozzle. Then, the interparticle cohesive force *f*_0_ (wall-particle and particle-particle friction) within the powder transfers the movement and the powder obtains a velocity υ. During the process of the acceleration decreasing to −∞, the actuator and the glass solid wall contract and stop moving, while the powder inside of the nozzle keeps moving forward a certain distance. That is to say, the powder inside the nozzle will obtain a pulse inertia force *F*_inertia_ relative to the solid wall of the glass nozzle. When the inertia force *F*_inertia_ is small in magnitude, the cohesive force *f*_0_*′* within the powder is greater than the resultant force of inertia force *F*_inertia_ and local gravity *G*_0_ of the powder itself, the powder will return with the nozzle, while, when *F*_inertia_ is large enough, the resultant force exceeds the cohesive force* f*_0_*′*, the powder will keep on moving forward to the orifice of the glass nozzle in the direction of the pulse inertia force. When the time of *t* is from 0 to *T*, the voltage slowly decreases from amplitude to zero. The produced acceleration of the lower surface of the actuator is a small value and the generated inertia force is not large enough to drive the movement of the powder relative to the nozzle. 

## 3. Results and Discussion

### 3.1. Aggregation Phenomena

**Figure 6:**
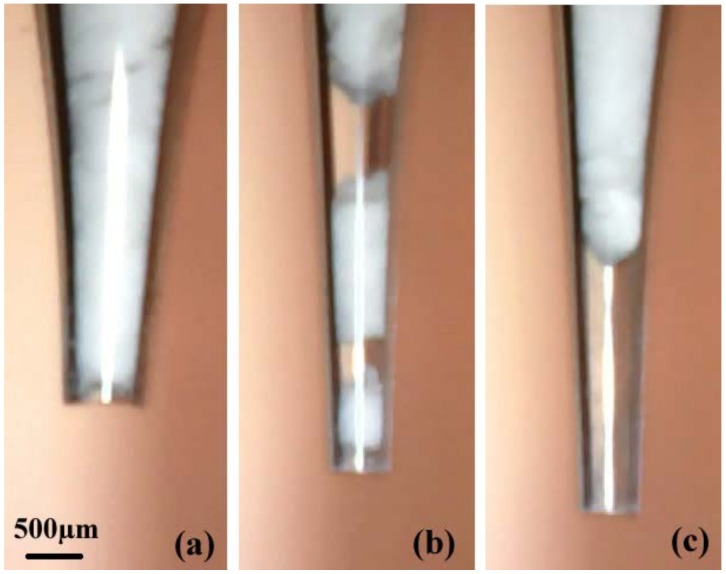
Aggregating phenomena of (**a**) arching, (**b**) plugging, and (**c**) blocking.

When the powder is loaded into the nozzle, the aggregation phenomena of arching, plugging, and blocking may occur, as is shown in Figure 6. Although Russo et al. [21] demonstrated that a kind of vibration technique was able to break particle aggregates when acting at the resonance frequencies of the aggregate structure (>100 Hz), this method cannot solve the aggregation problem in the micro-nozzle because the shape of the nozzle is tapered and a dense phase of powder is easily formed in the tapered section of the nozzle. In the arching structure, the powder occupies the whole tube and develops arches, preventing the powder from falling, as is shown in Figure 6a. In the plugging structure, a plug of powder and a bubble appear alternately. When the plug is discharged from the nozzle, the dosing mass is increased. When a bubble escapes from the nozzle, there is no mass increment, as is shown in Figure 6b. In the blocking structure, no powder is discharged from the nozzle and the micro-feeding process does not function, as is shown in Figure 6c. These powder structures in the nozzle cause the micro-feeding process to be unstable and inaccurate, and are strongly related to the powder cohesion and nozzle outlet diameter.

### 3.2. Micro-Dosing Mass

The micro-dosing mass is the most important parameter in the powder micro-dosing/feeding technology. The less the micro-dosing mass is, the higher the micro-dosing accuracy is. The micro-dosing mass is measured by an electronic balance and its resolution ratio is 0.1 mg. Besides that, the coefficient of variation (*C·V*) is used to research the micro-dosing stability. The coefficient of variation is calculated by:

(1) $$C\cdot V=\left(S/\bar{x}\right)100\%$$

where *S* denotes the standard deviation and $\bar{x}$ denotes the mean dose mass. The number of the samples is ten.

When being applied with a pulse voltage, the piezoelectric actuator expands and exerts a driving force on the solid wall of the glass nozzle. In consequence, the powder is discharged in a discrete mode and a small amount of powder will be discharged discontinuously out of the nozzle in a single pulse inertia force. The dose shape is a bar due to the high cohesive force among particles. The bubble of powder disappears from the nozzle and the powder occupies the whole outlet of the nozzle. The powder is compacted sufficiently and retains a stable structure. If the pulse inertia force is imposed on the nozzle and powder within it continuously, the powder at the nozzle tip will extrude as short bars which break off beyond the orifice after the bubble of powder disappears.

**Figure 7:**
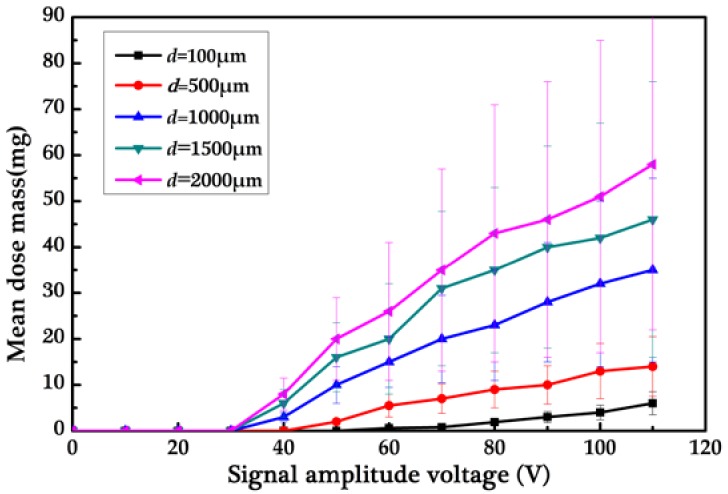
Variation of mean dose mass with voltage amplitude and nozzle outlet diameters.

**Figure 8:**
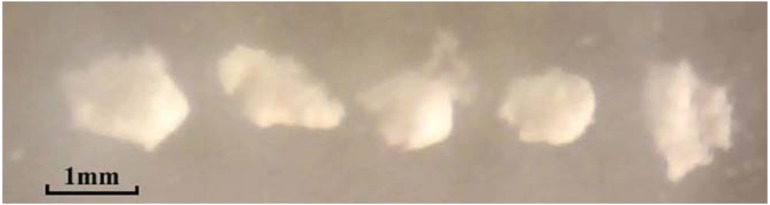
Dots of RespitoseSV003 monohydrate powder when the nozzle diameter is 500 μm.

Figure 7 shows that the mean dose mass increases with the voltages in different nozzle outlet diameters because the pulse inertia force inducing the amplitude of the dosing process is proportional to the signal amplitude voltage. In this experiment, the angle of the axis of the nozzle and vertical line is set as 0 degree. The frequency of the applied voltage (*f*) is set at a lower value of 1 Hz. Each data point is an average of ten measurements. The PZT stack actuator provides a greater pulse inertia force for powder inside when a higher signal amplitude voltage is applied with the help of powder gravity. Therefore, the powder will be discharged as short bars out of the nozzle orifice and the dose mass will increase when the voltage amplitude rises. If the voltage is low in magnitude, powder will not be discharged. For instance, the minimum voltage to discharge powder is 30 V for different sizes of nozzle orifice and the minimum mean dose mass is as small as 0.6 mg in a single pulse. On the other hand, when the voltage amplitude is relatively high, the inertia force is large enough to discharge a higher mass of powder. The mean dose mass is in the range of 0.6–58.0 mg in a single driving pulse. Figure 8 shows five discharged dots of RespitoseSV003 monohydrate powder, where the mean dose mass is about 10.0 mg when the nozzle diameter is 500 μm. Furthermore, the powder is not transported during the fast acceleration of the pipe (there is slip between powder and pipe) but during the slow motion (powder sticks onto the pipe and is thus driven in one direction). 

**Figure 9:**
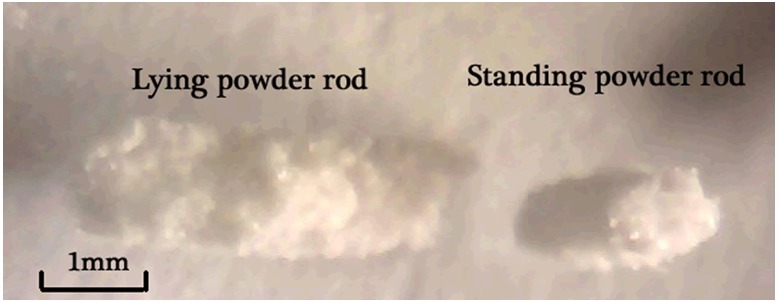
Cylindrical shape of discharged powder bars.

Table 2 records the data of stability experiments for powder pulse micro-dosing with the nozzle outlet diameter of 1 mm when the angle between the axis of the nozzle and vertical line is set as 0 degree. The coefficient of variation increases as the voltage also increases. The discharged powder bar is cylindrical in shape, as is shown in Figure 9. The upper/bottom circular diameters of the cylindrical shape are the same as each other, while the same size of nozzle is used and the diameter is approximately equal to the outlet diameter of the tapered glass nozzle. The length of the cylindrical shape is determined by the amplitude voltage. So, the higher the voltage is, the longer the length of the cylindrical shape is and the bigger the discharged powder bar is. In other words, the powder dose mass in a single pulse inertia force is determined by the breakup place of the cylindrical shape. When the voltage amplitude is relatively high, the randomness of the breakup place increases rapidly. So, the coefficient of variation is relatively high (above 15%) when the voltage amplitude is relatively high. 

**Figure 10:**
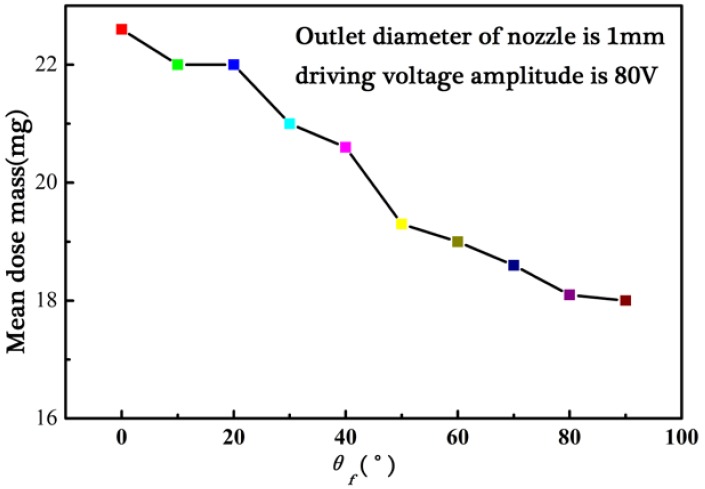
Variation of mean dose mass with the angle the axis of nozzle and vertical line.

The angle of the nozzle axis and vertical line (*θ*_f_) are important controlling parameters in the experiment with the nozzle outlet diameter of 1 mm and the voltage amplitude of 80 V, as is shown in Figure 10. The mean dose mass of RespitoseSV003 monohydrate powder decreases slowly with the increase of the angle of *θ*_f_. The dose mass is in the range of 18.0–22.6 mg. From the above analysis about the driving principle for powder actuated by the pulse inertia force, the value of the angle denotes the influence degree of local gravity *G*_0_ of the powder itself. That is to say, the local gravity *G*_0_ of the powder itself is a secondary factor in comparison to the pulse inertia force. The powder can be discharged from the nozzle orifice when the angle of the nozzle axis and vertical line is set as 90 degrees. The mean dose mass is about 18 mg for a driving pulse, which shows that the powder’s weight is one of the important factors instead of the decisive factor. The decision factor during the powder dose process is the pulse inertia force.

## 4. Conclusions

This paper presents a novel method for micro-doses of fine cohesive powders actuated by pulse inertia force. A piezoelectric actuator is used to provide a high enough pulse inertia force for a tapered glass nozzle. The cohesive powder is discharged out of the nozzle with the help of powder self-gravity. The α-lactose monohydrate powder is used as the micro-dosing powder. The dose mass can be controlled by changing any one of the tapered nozzle outlet diameter, amplitude of the applied pulse voltage, and angle of the nozzle axis and vertical line. The powder dose mass in a single pulse inertia force is determined by the breakup place of the ejected cylindrical shape of the fine powder bar. The mean dose mass increases with the increasing of the PZT stack actuator driving the signal amplitude voltage for different nozzle outlet diameters and the angle of the nozzle axis and vertical line has little influence on the dose mass. The minimum mean dose mass is as small as 0.6 mg in a single pulse inertia force. The coefficient of variation of the dose mass, which represents the micro-dosing stability, can be controlled below 5% when the dose mass is relatively small.

## Figures and Tables

**Table 1:** Properties of RespitoseSV003 monohydrate powder.

Name	Properties								
RespitoseSV003	Characteristics	X10/μm	X50/μm	X90/μm	Bulk density/g·cm^−3^	Tapped density	Carr Index	AoR	AR
	Sieved	46.1	71.0	100.0	0.67	0.85	20.8	35.85°	0.69

**Table 2:** Data of powder micro-dosing (mg) with the nozzle outlet diameter of 1 mm.

Samples	Voltage	40 V	50 V	60 V	70 V	80 V	90 V	100 V	110 V
1		3	9.3	15.2	17.6	22.6	23	29.3	29.2
2		3.2	10.6	14.1	21	21	26.5	34.2	30.5
3		3.1	10.9	16.2	23.3	23.5	31.8	28.3	38.3
4		2.9	11	15.9	18.3	19.3	29.5	39.1	39
5		2.8	10	17	19.6	25	31	30.5	41.6
6		2.9	10.2	15.6	21.4	24.5	32	32.2	31.2
7		3	10.6	14.7	17.3	18.3	24.5	26.2	39.5
8		3	9.9	14.6	22.2	22.6	30.2	36.3	36.7
9		2.8	10.6	13.2	22.2	18.5	24.6	36.8	35.3
10		3.1	9.2	16.2	19.5	24	26.2	24.3	26.4
$\bar{x}$		3.0	10.2	15.3	20.2	22.8	23	31.7	34.8
*C·V*		4.41%	6.13%	7.39%	10.32%	10.32%	12%	15.3%	14.69%
